# A postpartum vaccination promotion intervention using motivational interviewing techniques improves short-term vaccine coverage: PromoVac study

**DOI:** 10.1186/s12889-018-5724-y

**Published:** 2018-06-28

**Authors:** Arnaud Gagneur, Thomas Lemaître, Virginie Gosselin, Anne Farrands, Nathalie Carrier, Geneviève Petit, Louis Valiquette, Philippe De Wals

**Affiliations:** 1 Department of Pediatrics, Neonatology Unit, Faculty of Medicine and Health Sciences, Université de Sherbrooke, 3001 12e Avenue Nord, Sherbrooke, Quebec J1H 5N4 Canada; 2 Centre de Recherche du Centre hospitalier universitaire de Sherbrooke, 3001 12e Avenue Nord, Sherbrooke, Quebec J1H 5N4 Canada; 3 Eastern Townships Public Health Department, 300 King Est, bureau 300, Sherbrooke, Quebec J1G 1B1 Canada; 4 Department of Microbiology and Infectious Diseases, Faculty of Medicine and Health Sciences, Université de Sherbrooke, 3001 12e Avenue Nord, Sherbrooke, Quebec J1H 5N4 Canada; 5 Department of Social and Preventive Medicine, Laval University, Quebec City, Quebec Canada

**Keywords:** Motivational interviewing, Vaccine coverage, Infants, Health promotion intervention, Maternity wards

## Abstract

**Background:**

Due to the increasing number of vaccine-hesitant parents, new effective immunization promotion strategies need to be developed to improve the vaccine coverage (VC) of infants. This study aimed to assess the impact of an educational strategy of vaccination promotion based on motivational interviewing (MI) techniques targeting parents and delivered at the maternity ward, for the VC of infants at 3, 5, and 7 months of age.

**Methods:**

An individual educational information session, administered using MI techniques, regarding immunization of infants aged 2, 4, and 6 months was (experimental group) or was not (control group) proposed to parents during the postpartum stay at the maternity ward. Immunization data were obtained through the Eastern Townships Public Health registry for infants at 3, 5, and 7 months of age. Absolute VC increases at 3, 5, and 7 months in the experimental group were calculated and the relative risks with the respective 95% confidence intervals were computed using univariate logistic regression with the generalized estimating equations (GEE) procedure. Multivariate regression using GEE was used to adjust for confounding variables.

**Results:**

In the experimental and control groups, 1140 and 1249 newborns were included, respectively. A significant increase in VC of 3.2, 4.9, and 7.3% was observed at 3, 5, and 7 months of age (*P* < 0.05), respectively. The adjusted relative risk of the intervention’s impact on vaccination status at 7 months of age was 1.08 (95% confidence interval: 1.03–1.14) (*P* = 0.002).

**Conclusions:**

An educational strategy using MI techniques delivered at the maternity ward may be effective in increasing VC of infants at ages 3, 5, and 7 months. MI could be an effective tool to overcome vaccine hesitancy.

**Electronic supplementary material:**

The online version of this article (10.1186/s12889-018-5724-y) contains supplementary material, which is available to authorized users.

## Background

Vaccination is widely considered to be one of the greatest advancements in public health. Vaccination programs have contributed substantially to the decline in mortality and morbidity of infectious diseases with major public health importance [1, 2]. To be successful in reducing the prevalence and incidence of vaccine-preventable diseases, vaccination programs rely on high and sustained vaccine uptake [3–5]. Vaccination is not mandatory and is free of charge in Quebec. According to the most recent childhood National Immunization Coverage Survey (conducted every 2 years), vaccination uptake by vaccine type at age 2 years in 2014 varied from 71 to 85% [6]. This low level of vaccine coverage (VC) in childhood vaccination could be explained by the increasing number of parents who are ambivalent regarding the necessity and safety of vaccines, and it is possible that vaccination programs may be losing public confidence [7, 8].

Significant gaps in coverage in immunization programs have led to outbreaks of preventable diseases such as measles, whooping cough, rubella, and mumps, worldwide. For instance, in 2015, a large measles outbreak was traced to an unvaccinated traveler visiting Disneyland, which affected more than 20 US states, Mexico, and Canada, including 150 cases in an unvaccinated community in Quebec [9]. In addition, 13,726 cases of measles were reported between 1 November 2016 and 31 October 2017 in 30 European Union/European Economic Area member States, with the highest number of cases reported by Romania (5605) and Italy (4973). Among the cases with a known vaccination status, 87% were unvaccinated and 8% were vaccinated with a single dose, demonstrating that coverage failures may have contributed to outbreaks (https://ecdc.europa.eu/sites/portal/files/documents/Monthly-Measles-Rubella-monitoring-report-December-2017_0.pdf).

“Vaccine hesitancy” (VH) is a concept now frequently used in the discourse related to vaccine acceptance [10]. “VH” is described by the World Health Organization as the “delay in acceptance or refusal of vaccines, despite availability of vaccine services.” Vaccine-hesitant parents may accept with reluctance, delay or refuse one, some, or all vaccines [11].

Three effective interventions are known to increase vaccination uptake among populations: parent reminder and recall, multicomponent interventions that include education, and vaccination requirements for child care, school, and college attendance [12]. To date, there has been no evidence to suggest that education-only interventions were effective in improving VC [12]. Among the few studies that have addressed parental VH and refusal, no effective strategies have been proposed [13, 14]. With the increasing proportion of vaccine-hesitant parents, there is an important need to develop effective vaccination promotion strategies.

In this context, we developed an educational intervention program to be delivered at the maternity ward based on the motivational interviewing (MI) approach to promote early childhood immunization [15]. Described as a promising tool for the health promotion strategy [16], MI is a patient-centered communication style used to enhance the patient’s internal motivation for attitudinal change by exploring and solving inherent ambivalences [17]. Originally developed in the context of substance abuse, MI has also been applied for behavior change in several health-related fields such as nutrition, physical activity, and smoking cessation [18–20]. MI is based on four main principles: 1) empathizing with the client, 2) developing a discrepancy between their current and desired behavior, 3) dealing with resistance without antagonizing, preserving effective communication, and allowing clients to explore their views, and 4) supporting self-efficacy, i.e., the confidence in their ability to change [21]. The underlying spirit of MI is based on partnership, acceptance, compassion, and evocation. The goal is to engage the client in a collaborative working relationship, allowing the client to feel involved in the decision to change, in a respectful and non-judgmental atmosphere. Counseling based on MI involves five core communication skills: 1) asking open questions, 2) affirming, 3) reflective listening, 4) summarizing, and 5) informing and advising only if prior permission was given by the client.

An MI session delivered at a regional maternity ward in Quebec during the mother’s postpartum stay showed promising results with a significant 15% increase in the intention of mothers to vaccinate their infants at 2 months old [15]. The present study aimed to assess the impact of this novel educational strategy based on MI techniques, on the VC of infants at 3, 5, and 7 months of age.

## Methods

The methods section adheres to the Transparent Reporting of Evaluations with Non-randomized designs (TREND) statement checklist guidelines [22].

### Participants

This was a quasi-experimental cohort study using a static-group comparison design with multiple post-test measurements. It was conducted in the maternity ward of the *Centre hospitalier universtaire de Sherbrooke* (CHUS), located in the Eastern Townships region (Quebec, Canada). Births occurring at the CHUS represent 95% of the total births in the region. During a one-year period, eligible mothers (aged 18 or over, speaking French or English, and living in the Eastern Townships region) who gave birth at the CHUS and the respective newborn infants (twins included) were included in the study. Mothers or newborns requiring acute care were excluded from the study.

Mothers were screened during their postpartum stay in the maternity ward, over regular business hours (8AM to 5PM), in chronological order of delivery. In practical terms, this meant that mothers who had delivered first and who had not been approached by the research team were screened first. This approach was adopted in order to optimize recruitment given the short duration of postpartum maternity ward stays (mean duration = 48 hours). Mothers who agreed to participate signed an informed consent form.

All children whose mothers received the study intervention were assigned to the experimental group, while the static control group included children of mothers who were not approached to participate in the study. Two other groups were considered in this study: the primary refusals group, with children of approached mothers who refused to participate in the study, and the secondary refusal or impossible intervention group comprising children of mothers who agreed to participate but withdrew their consent before receiving the intervention because of fatigue or breastfeeding issues.

### Intervention

The MI-based educational intervention followed the Quebec Immunization Protocol [23] and was developed according to the study conceptual framework [15]. Using an MI-specified empathic communication style, it addressed several pieces of information such as the 6 vaccine-preventable diseases (VPD) at 2, 4, and 6 months of life, the effectiveness of vaccines, the importance of the routine immunization schedule, and the fears and side effects associated with vaccination. During 2010 to 2011, the Quebec routine immunization schedule recommended two vaccines at 2, 4, and 6 months of age to protect against diphtheria, tetanus, poliomyelitis, whooping cough, and *Haemophilus influenzae* type b (Hib), and pneumococcus infections [23]. The intervention content was adapted from two existing theoretical frameworks: 1) the Health Belief Model [24] and 2) the transtheoretical model of behavior change [25]. Based on this composite model, the intervention was adapted to each participating family according to the current intention of the parent to vaccinate his/her newborn at 2 months of age.

The MI session was administered once individually to consenting mothers 24 to 48 hours after the delivery in their rooms by one of 3 clinical research assistants, who had received standardized training on the intervention’s content and MI techniques. The intended duration of the intervention was 20 minutes (Additional file 1).

### Objectives

We hypothesized that an individualized educational information session regarding immunization and given during postpartum hospitalization would improve VC for the 2-, 4-, and 6-month vaccines. The aim of the study was to assess the impact of this novel educational strategy based on MI techniques, on the VC of infants at 3, 5, and 7 months of age.

### Outcomes

The main outcome measures were the VC of infants at 3, 5, and 7 months of age. The secondary outcome measure was the feasibility rate of the intervention (i.e., the proportion of mothers who received the intervention/mothers who accepted to receive the intervention).

To evaluate the VC of infants, vaccination data were obtained from LOGIVAC, the immunization registry of the Eastern Townships region. This exhaustive registry contains all births that have occurred in the region and records all vaccines administered to residents of the Eastern Townships since 1998, including data for those born outside the region. Thus, all children born in the region, regardless of their vaccination status, are included in the LOGIVAC registry. Vaccination data were extracted by the Eastern Townships Public Health Department for all the participant infants (experimental group), children of mothers who were not approached (control group), and for children of mothers who refused the intervention (primary and secondary refusal groups). Because we had access to nominal data for all the mothers who gave birth at the CHUS, the extraction of vaccination data of infants for all eligible mothers in the study was possible. The extraction of nominal vaccination data and data pairing with the study data was performed by a research agent of the Eastern Townships Public Health Authority, who was not involved with our study and was blinded to the assignation groups of the study.

### Determination of the immunization status

The immunization status was determined at 3, 5, and 7 months of age for each eligible infant in order to allow a reasonable time to receive the recommended vaccines at 2, 4, and 6 months, as included in the routine immunization schedule. This one-month delay to assess VC corresponds to the national standards established by the Canadian Immunization Registry Network [26]. A child was considered to have a complete vaccination status if he/she received all vaccines or antigens recommended by the Quebec Immunization Protocol, except for the vaccines against influenza viruses [23].

### Assignment method and sample size

Once the immunization status was determined for all children, the main outcome measures (VC at 3, 5, and 7 months) were computed for each study group as the proportion of children with a complete vaccination status among the total number of children in each group. The independent variables, such as mother’s age, length of postpartum hospitalization, cesarean birth, infant’s rank in the family, and hospitalization of the newborn in the neonatology ward during the postpartum stay, were used to assess the comparability of groups and to control for potential confounding factors. In order to identify a statistically significant amelioration of 5% in the VC of infants, and taking into account a VC of 80% [6], a risk of alpha error of 0.05 and a power of 80%, a total of 943 mothers per group should be recruited accordingly with the 3000 annual births at the maternity ward of the CHUS.

### Data analysis

Characteristics of included mothers and newborns were analyzed using descriptive statistics such as frequency, mean and standard deviation (SD), and median and interquartile range (IR). These characteristics were compared between experimental and control groups using the χ^2^ test for dichotomized variables, and the Student’s *t*-test or Mann-Whitney test for continuous variables both normally and not normally distributed, respectively. Respective VC at 3, 5, and 7 months of age was compared between experimental and control groups using χ^2^ tests to assess the impact of the intervention. Relative risks (RR) with respective 95% confidence intervals (CIs) were computed for each VC using univariate logistic regressions according to the generalized estimating equations (GEE) procedure. Both per-protocol (PP) and intention-to-treat (ITT) analyses were performed. Furthermore, multivariate regressions with the GEE procedure were realized in the PP and ITT analyses to assess the intervention’s impact on VC adjusted for mother’s age, cesarean birth, birth order, and hospitalization in the neonatology ward. Finally, in order to ascertain the absence of selection bias, the VC at ages 3, 5, and 7 months of the three study groups who did not receive the intervention were compared using χ^2^ tests. Statistical analyses were performed using IBM SPSS version 20.0 (Armonk, NY) and SAS version 9.3 (SAS Institute Inc., Cary, NC, USA), with statistical significance set at 0.05.

### Ethical considerations

Approval for this study was obtained from the CHUS Ethics Committee in Humans Health Research. In addition, we obtained an authorization from the “Commission d’accès à l’information du Québec” (CAI) to have access to vaccination data contained in the immunization registry.

## Results

### Study participants

**Fig.1:**
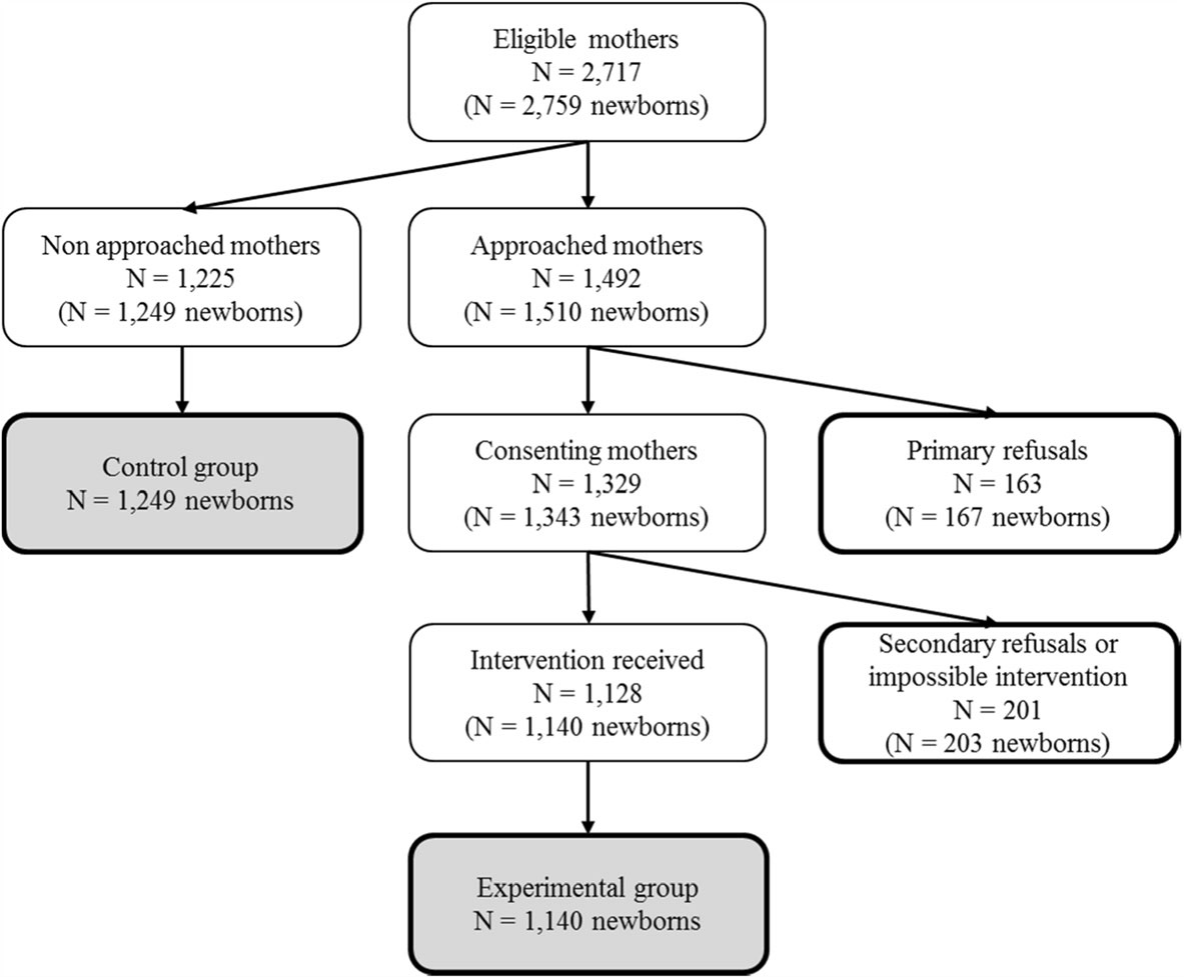
Study flow chart

During the study period, 1128 mothers agreed to participate and received the MI session. The corresponding newborns (1140, including 12 twins) were assigned to the experimental group (Fig. .1). The control group included 1249 newborns of mothers who were not approached to participate in the study. Children whose mothers refused to take part in the study constituted the “primary refusal group” for a total of 167 newborns while children whose mothers agreed to participate but withdrew their consent or were not available to receive the intervention constituted the “secondary refusal or impossible intervention group” for a total of 203 newborns. These 203 children were included in the experimental group for the ITT analysis. Thus, 1140 and 1343 newborns were included in the experimental group in the PP and ITT analyses, respectively.

There was no statistically significant difference in the mothers’ ages and the length of postpartum hospitalization between the experimental and control groups in the PP analyses (Table 1). However, statistically significant differences were observed for the following variables: “*At least one another child in the family,*” “*Cesarean birth,*” and “*Newborn hospitalized in the neonatology ward during postpartum stay.*” Similar results were obtained by ITT analyses, except for “*Cesarean birth,*” for which non-significant differences were observed.

**Table 1:** Characteristics of the participating families

	Experimental group *n*=1,140	Control group *n*= 1,249	*p-value*
Mothers’ age, years, mean ± SD	28.4 ± 5	28.5 ± 5	.593
Length of postpartum hospitalization, hours, med (IQR)	48 (48-72)	48 (48-72)	.328
At least one another child in the family, n (%)	587 (51.5)	725 (58.0)	.001
Caesarean birth ^a^, n (%)	187 (16.4)	267 (21.4)	.002
Newborn hospitalized in the neonatology ward during postpartum stay, n (%)	51 (4.5)	172 (13.8)	<.001

^a^ Two missing values in the experimental group and one missing value in the control group

### Feasibility of the intervention

Of the 1329 mothers who initially agreed to receive the intervention, 115 later refused to attend the educational session and 86 could not be reached during the postpartum stay. The feasibility rate of the educational session was 93% (1243/1329 mothers).

### Intervention’s impact on the VC of infants

The MI session significantly increased the VC of infants by 3.2, 4.9, and 7.3% (Table 2), and 2.7, 3.8, and 5.7% (Table 3), at 3, 5, and 7 months of age in the PP and ITT analysis, respectively. After adjustment for the characteristics of the mothers and the newborns, the estimated RR of the intervention’s impact on vaccination status at 7 months of age was 1.08 (1.03–1.14) (*P* = 0.002) and 1.07 (1.02–1.12) (*P* = 0.009), in the PP and ITT analyses, respectively (Table 4).

**Table 2:** Intervention’s impact on VC at 3, 5 and 7 months (PP analysis)

	Experimental group *n*=1,140	Control group *n*= 1,249	Absolute difference of VC (%)	RR (95% CI)	*p-value*
Complete VC at 3 months	1,041 (91.3)	1,101 (88.1)	3.2	1.04 (1.01-1.06)	.011
Complete VC at 5 months	948 (83.2)	978 (78.3)	4.9	1.06 (1.02-1.10)	.003
Complete VC at 7 months	865 (75.9)	857 (68.6)	7.3	1.11 (1.05-1.16)	<.001

**Table 3:** Intervention’s impact on VC at 3, 5 and 7 months (ITT analysis)

	Experimental group *n*=1,343	Control group *n*= 1,249	Absolute difference of VC (%)	RR (95% CI)	*p-value*
Complete VC at 3 months	1,220 (90.8)	1,101 (88.1)	2.7	1.03 (1.00-1.06)	.025
Complete VC at 5 months	1,103 (82.1)	978 (78.3)	3.8	1.05 (1.01-1.09)	.014
Complete VC at 7 months	999 (74.3)	857 (68.6)	5.7	1.08 (1.03-1.14)	0.001

**Table 4:** Adjusted intervention’s impact on completed vaccination status at 7 months old (PP and ITT analyses)

	PP Analyses		ITT Analyses	
Variables	Adjusted RR (95% CI)	*p-value*	Adjusted RR (95% CI)	*p-value*
Intervention	1.08 (1.03-1.14)	0.002	1.07 (1.02-1.12)	.009
Mother's age	1.00 (0.99-1.01)	0.083	1.00 (0.99-1.01)	.201
Caesarean birth	0.98 (0.92-1.05)	0.642	0,97 (0.91-1.03)	.269
At least one another child in the family	0.89 (0.85-0.93)	<0.001	0.88 (0.83-0.92)	<.001
Hospitalization in the neonatology ward during postpartum stay	0.89 (0.80-0.99)	0.027	0.91 (0.83-1.01)	.066

### Monitoring of selection bias

There was no statistically significant difference between the children’s VC by mothers who did not receive the study intervention (control group, primary refusals group, and secondary refusals or impossible intervention group).

## Discussion

This regional study was the first to assess the impact of a brief MI session during the postpartum stay on the short-term VC of infants. Our findings confirm our previous results, which revealed an increase of 15% in vaccine intention of mothers who received the intervention, and a significant increase in VC in infants [15].

However, due to study design limitations, conclusions should be made carefully. This study used a quasi-experimental design and was not a randomized control trial. The aim of this study was to assess the feasibility and the potential impact of the MI strategy in a regional cohort to conduct a future randomized controlled trial in case of positive results. The study limitations were attributed to the characteristics of the experimental and control groups, which were similar except for the proportion of cesarean births and the proportion of hospitalizations in the neonatology ward (these were higher in the control group because mothers or newborns requiring acute care were excluded from the study). The control group also showed a higher proportion of mothers with newborns that were not their first children. This difference between the two groups may have had an impact on the results of this study because we previously showed that the intention to vaccinate an infant at 2 months old was positively associated with having at least one child. Thus, the control group may have had a stronger intention to vaccinate than did the experimental group, which could have minimized the effects of the educational session. Moreover, as consenting mothers were not randomized to receive the intervention, there was no significant difference in the VC of children whose mothers did not receive the intervention (control group, primary refusals group, and secondary refusals or impossible intervention group), suggesting the absence of a selection bias. In addition, the immunization registry did not contain vaccination data for children who moved out of the region after their birth. Thus, the VC calculated in this study might be underestimated. However, this bias was non-differential, as relocations could have occurred both in the experimental and the control groups. Despite some improvements in the reminder and recall procedures by the Eastern Townships Public Health Department, the impact of the MI session may be considered to have resulted in the observed increase in the experimental group because reminders and recalls were likely addressed to both groups (experimental and control).

Despite the above limitations, the results showed that the MI session was effective, as a sustained increase (from 3.2 to 7.3%) in the VC of infants at 3, 5, and 7 months old was observed. Results of the ITT analysis were similar and also statistically significant, strongly reflecting the effectiveness of the study intervention. Moreover, the improvement in the VC observed at 7 months was significant even after adjusting for confounding variables. Indeed, the results showed that infants of mothers who received the MI session had an 8% higher chance of having a complete vaccination status at the age of 7 months than did children whose mothers did not receive the intervention. These improvements in the short-term infant VC are among the best reported in the literature [12, 13], and are highly encouraging, considering that delays in the first vaccination at 2 months old are associated with a higher probability of having an incomplete immunization status at 2 years of age [5, 27–32]. The significant increase in VC at 3, 5, and 7 months could thus predict an increase in VC for all childhood vaccines. Furthermore, this study confirmed our previous results showing an increase of 15% in the intention to vaccinate among mothers who received the MI session [15]. This indicates that the mother’s intention to vaccinate and immunization behavior are highly associated.

Traditional education methods have not been effective in addressing VH [33], while some studies have shown that attempting to convince vaccine-hesitant parents to vaccinate their child by giving them more facts may be counterproductive and increase hesitancy [34]. On the contrary, the high level of acceptability and effectiveness of the PromoVac strategy could be related to the use of the MI techniques. Indeed, the use of the MI approach facilitates a respectful and empathetic discussion of concerns about vaccinations, and helps to build a strong relationship between the parents and the nurses. Parents are given an opportunity to talk freely about their concerns and ask questions about vaccinations without feeling judged. The intervention is adapted to the parents’ needs, based on their own concerns and questions, and therefore avoids giving them unnecessary or unwanted information. The nurses were able to help parents to explore their own ambivalence and to help them find their own arguments for change in order to make an informed decision about their child’s vaccination. Our study is the first to demonstrate the potential effectiveness of an immunization promotion strategy using the MI approach in the field of vaccination. Only two studies have used the MI techniques to promote immunization among adults [35, 36]. Although their results were promising, they were not significant, mostly because of the small sample size [36] and the specific targeted population (adults undergoing methadone maintenance treatment) [35]. A good communication strategy involves understanding people, establishing a respectful partnership and helping them to modify their behavior according to their capacities.

A major strength of this study was the availability of vaccination data from a regional immunization registry, allowing the study of the intervention’s impact on VC of a large birth cohort of nearly 3000 newborns. Moreover, as 95% of the deliveries in the Eastern Townships occurred at the maternity ward of the CHUS, our sample was representative of the total population of mothers of newborns in the region. Moreover, according to the Health Insurance Plan in Quebec, hospitalization of mothers during childbirth is free of charge and does not motivate mothers’ choice to give birth at home or in hospital. However, mothers who gave birth at home or in birth-houses were not included in the study. These mothers may have different opinions regarding vaccination and may have a higher tendency to not have their children immunized.

## Conclusions

This regional study following a large birth cohort is the first to demonstrate the effectiveness of a brief educational intervention using the MI approach during the postpartum stay. In addition to an increase of 15% in the mothers’ intention to vaccinate their child, the intervention also significantly increased the VC of infants at 7 months old by 7%. A forthcoming study will assess the intervention’s impact on long term VC. This study presents a proof of concept of the MI intervention, and a randomized controlled trial may thus be conducted to validate these results. The need for effective strategies to tackle VH is critical. We suggest that MI may represent one of the most promising avenues of vaccination promotion strategies.

## Additional file

Additional file 1: Supplementary Material - Intervention tool. (DOCX 32 kb)

## Abbreviations

CAI: *Commission d’accès à l’information*
CHUS: Centre hospitalier universitaire de Sherbrooke
CI: confidence intervals
GEE: Generalized estimating equations
IR: Interquartile range
ITT: Intention-to-treat
MI: Motivational interviewing
PP: Per-protocol
RR: Relative risk
SD: Standard deviation
VC: Vaccine coverage
VH: Vaccine hesitancy
VPD: Vaccine-preventable diseases
