# Supplementary material for: A postpartum vaccination promotion intervention using motivational interviewing techniques improves short-term vaccine coverage: PromoVac study
Source: BMC Public Health. 2018 Jun 28;18:811. doi: 10.1186/s12889-018-5724-y (PMC6022497; doi:10.1186/s12889-018-5724-y)
Supplement: Supplementary file 1 — Supplementary Material - Intervention tool. (DOCX 32 kb) [file 12889_2018_5724_MOESM1_ESM.docx]

**Supplementary Material - Intervention tool**

*General Principle*

The intervention proposed in this study is an educational strategy using motivational interviewing (MI) techniques to educate parents of newborns about vaccination. Based on the MI approach, this intervention is carried out in simple and understandable language in order to foster discussion and allow questions from parents rather than provide prescriptive, unequivocal and unidirectional information. The MI intervention was developed according to Prochaska’s stages of change (see below) and lasts approximately 15 to 20 minutes.

*Motivational Interviewing*

MI is a brief intervention style that proposes a client-centered approach used to enhance the client’s internal motivation to change by exploring and solving their own ambivalence. It first aims to help individuals to make a decision and elicit and strengthen their motivation to change behavior based on the person’s own arguments for change. MI is based on four main intervention strategies to help clients:

1. Expressing empathy: MI emphasizes understanding and acceptance of the client’s knowledge, attitudes and experiences rather than the physician’s role as expert.
2. Developing discrepancy between the client’s current and desired behaviour (between the current situation and what the person wants).
3. Rolling with resistance and not against it to prevent communication rupture and allowing them to explore their views.
4. Supporting self-efficacy, i.e. the confidence in their ability to change.

*Prochaska’s Stages of Change*

Prochaska and DiClemente have proposed a unified theory defining several stages in which persons find themselves when they want to change their behavior. This classification can assist Health care professionals in developing appropriate arguments to induce people to modify their behavior. Prochaska’s cycle defines several stages of behavior change:

1. The pre-intention or pre-contemplative stage: the person does not consider change. They do not plan to change their behavior in the next six months. This may be explained by a host of reasons, that must all be taken into consideration.
2. The intention or contemplative stage: the person is ambivalent about change. They envisage modifying their habits in the relatively near future. The person weighs the pros and cons. The problem is defined, but action must be taken.
3. Preparation or determination: the decision is made and the person prepares for change. The practical arrangements for the change are defined.
4. Action: the period during which the person actually changes his/her habits. This is an important moment that requires a lot of energy and attention.

These stages of change can be adapted to the position of the parents toward the initiation of the vaccination of their child in the following way:

1. The **undecided** parent (first 2 stages), either at the **pre-contemplation stage** (the parent is resistant to vaccination) or at the **contemplation stage** (the parent is starting to reflect positively on vaccination but is still hesitant);
2. The **committed parent** (last 2 stages), either at the **preparation stage** (the parent is in favor of vaccination) or at the **action stage** (the parent takes concrete actions towards vaccination).

In order to mobilize the parent toward immunization, it is important to approach them at the stage that best defines their current intention to vaccinate their child. The aim of the intervention is to bring the parent to a higher stage and not to the final decision to vaccinate. It is fundamental to bear in mind that changing opinion on immunization is not an easy process.

*Description of the intervention*

Before starting the intervention, the research assistant sets out to define the position of the parent toward vaccination according to Prochaska’s stages, using 2 questions:

1. “The purpose of today’s discussion is to talk about the vaccination of [name of the child]. Overall, what is your opinion, your perception of vaccination?” This allows the research assistant to classify the parent as an undecided or committed parent.

2. “Regarding the vaccination of [name of the child], have you made your decision? Do you plan to vaccinate [name of the child]?” This confirms the position of the parent. The research assistant accepts the response without judgment and adapts the intervention according to the parent’s position.

At this stage of the intervention, five information points are discussed, using MI techniques, in the order and at the level presented in Table 1, according to the parent’s stage of change:

Step 1) Vaccine-preventable diseases targeted by the first vaccine series and their consequences;

Step 2) Vaccines and their effectiveness;

Step 3) Importance of the immunization schedule;

Step 4) Reluctance and adverse effects of vaccination;

Step 5) Organization of vaccination services.

| **Prochaska’s Stages of Change** | **PRE-CONTEMPLATION** | **CONTEMPLATION** | **PREPARATION** | **ACTION** |
| --- | --- | --- | --- | --- |
| **Level of Vaccine Hesitancy** |  | | | |
| **Vaccination Intention** | **NOT READY**  Parents are resistant to vaccines. | **GETTING READY**  Parents hesitate to vaccinate (ambivalence). They often have a lot of fears/concerns. | **GETTING READY / READY**  Parents want to vaccinate but may still have some fears/concerns (some ambivalence). | **READY**  Parents want to vaccinate and they know how to proceed toward vaccination. |
| **Possibilities of the intervention** | Confirm parents’ position | Confirm parents’ position | Confirm parents’ position  Congratulate them | Confirm parents’ position  Congratulate them |
|  | What are the disadvantages of vaccination?  What are your fears/concerns? | What are your fears/concerns? | Could there be barriers to vaccination?  Do you have any fears/concerns? | Could there be barriers to vaccination?  Do you have any fears/concerns? |
|  | **Step 4 +++** | **Step 4 +++** | **Step 4 if needed** | **Step 4 if needed** |
|  | Do you see any benefit from vaccination? | Do you see any benefit from vaccination? |  |  |
|  | **INFORMATION** | **INFORMATION** | **ORGANIZATION** | **ORGANIZATION** |
|  | **Step 1 and 2 ++** | **Step 1 and 2 ++** | **Step 5 +++** | **Step 5 +++** |
|  | **Step 3 +** | **Step 3 +** | **INFORMATION** | **INFORMATION** |
|  | If you decide to vaccinate: | If you decide to vaccinate: | **Step 1 ++ and 3 +++** | **Step 1 ++ and 3 +++** |
|  | **Step 5 ±** | **Step 5 ±** | **Step 2 +** | **Step 2 +** |
